# Biphasic Role of Chondroitin Sulfate in Cardiac Differentiation of Embryonic Stem Cells through Inhibition of Wnt/β-Catenin Signaling

**DOI:** 10.1371/journal.pone.0092381

**Published:** 2014-03-25

**Authors:** Robert D. Prinz, Catherine M. Willis, Toin H. van Kuppevelt, Michael Klüppel

**Affiliations:** 1 Ann and Robert H. Lurie Children’s Hospital of Chicago Research Center, Chicago, Illinois, United States of America; 2 Department of Pediatrics, Feinberg School of Medicine, Northwestern University, Chicago, Illinois, United States of America; 3 Robert H. Lurie Comprehensive Cancer Center, Feinberg School of Medicine, Northwestern University, Chicago, Illinois, United States of America; 4 Radboud Institute for Molecular Life Sciences, Department of Biochemistry, Nijmegen, Netherlands; University of California, San Diego, United States of America

## Abstract

The glycosaminoglycan chondroitin sulfate is a critical component of proteoglycans on the cell surface and in the extracellular matrix. As such, chondroitin sulfate side chains and the sulfation balance of chondroitin play important roles in the control of signaling pathways, and have a functional importance in human disease. In contrast, very little is known about the roles of chondroitin sulfate molecules and sulfation patterns during mammalian development and cell lineage specification. Here, we report a novel biphasic role of chondroitin sulfate in the specification of the cardiac cell lineage during embryonic stem cell differentiation through modulation of Wnt/beta-catenin signaling. Lineage marker analysis demonstrates that enzymatic elimination of endogenous chondroitin sulfates leads to defects specifically in cardiac differentiation. This is accompanied by a reduction in the number of beating cardiac foci. Mechanistically, we show that endogenous chondroitin sulfate controls cardiac differentiation in a temporal biphasic manner through inhibition of the Wnt/beta-catenin pathway, a known regulatory pathway for the cardiac lineage. Treatment with a specific exogenous chondroitin sulfate, CS-E, could mimic these biphasic effects on cardiac differentiation and Wnt/beta-catenin signaling. These results establish chondroitin sulfate and its sulfation balance as important regulators of cardiac cell lineage decisions through control of the Wnt/beta-catenin pathway. Our work suggests that targeting the chondroitin biosynthesis and sulfation machinery is a novel promising avenue in regenerative strategies after heart injury.

## Introduction

The glycosaminoglycan chondroitin sulfate (CS) consists of linear chains of repeating disaccharide units covalently linked to cell surface and secreted proteins to form chondroitin sulfate proteoglycans [1], [2], which have been shown to control multiple aspects of cellular behavior and communication [2]. Differentially sulfated CS forms include the mono-sulfated chondroitin-4-sulfate (C4S) and chondroitin-6-sulfate (C6S) units, as well as the di-sulfated units chondroitin sulfate-D (CS-D) and chondroitin sulfate-E (CS-E) [2], [3]. CS biosynthesis and its sulfation balance is tightly controlled by growth factor signaling [2], [4], [5], and in turn can control cellular signaling pathways [6], [7], [8], [9], [10]. Moreover, chondroitin sulfates have been functionally linked to various human diseases, including cancer, osteoarthritis, malaria, and others [2], [11], [12], [13], [14], [15], [16]. In contrast, more knowledge is required in regards to the importance of chondroitin sulfate molecules and sulfation patterns during mammalian development and cell lineage specification. Some of the better-known functions of chondroitin sulfates are in neural [17], [18] and skeletal [2] development and disease. Chondroitin sulphate proteoglycans are key modulators of spinal cord and brain plasticity [18], and are important molecular targets in therapies for spinal cord injuries [19]. Chondroitin-4-sulfation negatively regulates axonal guidance and growth in mice [20], and the regulation of a neuronal phosphoproteome by chondroitin sulfate proteoglycans has been described [9]. Moreover, CS plays roles in the control of signaling pathways essential for the proliferation, self-renewal, and cell lineage commitment of neural stem/progenitor cells [21]. We have previously described severe embryonic skeletal abnormalities and perinatal lethality in mice carrying a loss-of-function mutation in the *Chondroitin-4-sulfotransferase-1* (*C4st-1*) gene [7], demonstrating the critical importance of a proper balance of chondroitin sulfation in cartilage development. These conclusions were supported by other studies, which also identified skeletal abnormalities in loss-of-function mutations of a number of CS biosynthesis enzymes [22], [23]. Moreover, missense mutations in the human *Carbohydrate sulfotransferase 3* (*CHST3*) gene, involved in the production of CS, have been shown to be associated with Larsen syndrome, humero-spinal dysostosis [24], and Omani-type spondyloepiphyseal dysplasia [25], all of which cause severe skeletal abnormalities. Taken together, the literature clearly identifies a critical role for CS in mammalian neuronal and skeletal development and disease. Recently, mild cardiac abnormalities, including mitral, tricuspid and aortic regurgitations, have been described in a subset of Omani-type spondyloepiphyseal dysplasia patients, but not in patients with Larsen syndrome or humero-spinal dysostosis [26]. In contrast, *CHST3* knock-out mice and have neither skeletal nor cardiac defects [27]. Distinct expression domains for CS and CS biosynthesis enzymes have been described in the developing and mature mammalian heart [4], [28], [29]; however, the functional roles of CS in heart development or cardiac lineage development are not understood.

The Wnt/beta-catenin signaling pathway plays critical roles in many developmental processes, and aberrant Wnt/beta-catenin pathway activity is causally associated with many human diseases, including cancers [30], [31], [32], [33], [34], [35], [36], [37]. Wnt/beta-catenin signaling also controls stem cell behavior, for example in the intestinal epithelium [37], [38], [39], [40], [41], [42] Wnt/beta-catenin signaling also plays critical roles in embryonic stem (ES) cell renewal and lineage determination [43]. In cardiac lineage development, Wnt/beta-catenin has been shown to play a biphasic role [44], [45], [46]. At early stages, pathway activity is required for mesoderm formation, induction of precardiac mesoderm, and for the expansion of cardiac progenitor cell. At later stages, Wnt/beta-catenin signaling appears to inhibit the differentiation of cardiac progenitor cells into functional cardiomyocytes [44], [45], [46].

Interestingly, CS has recently been shown to control the Wnt/beta-catenin pathway. CS-E, but not other CS forms, can bind Wnt3a ligand with high affinity [47]. We recently demonstrated in NIH3T3 cells that treatment with CS-E could reduce activation of Wnt3a-receptor complexes on the cell surface, and limits Wnt/beta-catenin signaling to a threshold level of approximately 25% [8]. This threshold differentially affected transcriptional and biological readouts of Wnt/beta-catenin pathway activation [8]. Several studies have demonstrated a correlation of Wnt/beta-catenin signaling levels with embryonic stem cell differentiation, anterior specification during mouse embryogenesis, adult hepatic homeostasis, phenotypic severity of intestinal tumorigenesis, and lineage determination during hematopoiesis [30], [31], [32], [33], [34], [35], [36], [37], [42], [43]. Together, these results might suggest that CS and the balance of chondroitin sulfation could play a role in establishing critical Wnt/beta-catenin signaling thresholds in development and disease.

Here, we initially set out to investigate the roles of CS in ES cell differentiation in embryoid body (EB) cultures. We demonstrate by lineage marker analysis that enzymatic elimination of endogenous chondroitin sulfate by the bacterial enzyme Chondroitinase ABC (ChABC) leads to a defect in cardiac differentiation, and causes a reduction in the number of functional cardiac foci. We further show that elimination of CS in EB cultures activates the Wnt/beta-catenin pathway, a known regulatory pathway with a biphasic function in cardiac lineage determination. Limiting ChABC treatment to only early or late stages of ES cell differentiation mimics the biphasic effects of Wnt/beta-catenin pathway activation on cardiac differentiation. Temporally restricted treatment with a specific exogenous chondroitin sulfate, CS-E, inhibits Wnt/beta-catenin signaling and phenocopies the biphasic effects of endogenous CS on cardiac differentiation and Wnt/beta-catenin signaling. Together, these results establish a novel biphasic role of chondroitin sulfate in the specification of the cardiac cell lineage during embryonic stem cell differentiation through inhibition of the Wnt/beta-catenin pathway.

## Materials and Methods

### Reagents

Chondroitinase ABC (protease-free) and CS-E were obtained from The Associates Of Cape Cod, USA. C4S was obtained from Sigma Inc., USA.

### ES Cell and Embryoid Body (EB) Culture

R1 ES cells and EBs were cultured as previously described [4]. Briefly, R1 ES cells were cultured on gelatinized tissue culture plates in ES media containing Leukemia Inhibitory factor (LIF; Gibco, USA). In experiments requiring LIF withdrawal, ES cells were cultures for 6 days in the absence of LIF. For EB culture, ES cells were trypsinized and plated on 24-well low cluster plates (Costar, USA) in ES media without LIF. After 4 days, EBs were transferred to gelatinized 12-well or 24-well tissue culture plates for flat culture for an additional 8 days. Bi-daily treatments of EBs included ChABC (10 mU/ml), CS-E (100 microgram/ml), or C4S (100 microgram/ml), as previously described [8], [48].

### Immunofluorescence Microscopy

Immunofluorescence microscopy was performed as previously described [8]. Briefly, ES cells were grown on gelatinized glass slides (Biotek, USA). Following fixation in 4% PFA/PBS for 5 minutes and permeabilization in 0.1% Triton X100 for 2 minutes, cells were blocked in 10% FBS/PBS, followed by over night incubation with primary antibodies mouse anti-C4S or rabbit anti-b-catenin (Santa Cruz Biochemicals, USA). This was followed by a one-hour incubation with donkey anti-mouse or anti-rabbit Alexa-Fluor-488 secondary antibody (Invitrogen, USA). DAPI was used to counterstain nuclei, followed by mounting in Prolong Gold Antifade (Invitrogen, USA). For proliferation assays, the Click-iT EdU cell proliferation assay (Life Technologies, USA) was used.

### mRNA Preparation and Quantitative Real Time PCR (qPCR)

RNA was prepared using TRI Reagent or TRIZOL according to the manufacturer’s instruction. Subsequently, 1.5 microgram of RNA were reversed transcribed using MuMLV-Reverse Transcriptase (Promega, USA), followed by real time amplification using a 2xSYBR Green PCR Master Mix (Applied Biosystems, USA, Fisher Scientific, USA, or Biotium, USA) on an Applied Biosystems 7500 Real Time PCR platform in 15 microliter reactions using an annealing temperature of 60°C.

The following primer pairs were used (5′ to 3′):

HPRT:

ATGCCGACCCGCAGTCCCAGC and CGAGCAAGTCTTTCAGTCCTGTCC

Oct4:

TTGGGCTAGAGAAGGATGTGGTT and GGAAAAGGGACTGAGTAGAGTGTGG

Nanog:

GTGCTGAGCCCTTCTGAATC and GAACTCTCCTCCATTCTGAAC

Brachyury:

CTGGCCACACCAGCATGCTGCC and GTACCATTGCTCACAGACCAGAGACTG

Mesogenin:

CCGGGATCCTGGGTGAGACCTTCCTCAGC and TGCCAAGCTTGGCCTGGGCTCTCTCCCGC

Fgf5:

GATTGTAGGAATACGAGGAGTTTTCAGCAAC and TCTTGGAATCTCTCCCTGAACTTACAGTCA

Afp:

AGCAAAAGCCTGAACTGACAGAGGAGCAG and GTTTTGGAAATCAACTTTGGACCCTCTTCTGTG

Gata4:

AAGAGATGCGCCCCATCAAGACA and TGGGGACAGCTTCAGAGCAGACAG

Nkx2.5:

GGCGTCGGGGACTTGAACACC and CGCACTCACTTTAATGGGAAG

Mlc-2v:

TGGGTAATGATGTGGACCAA and GGGAGGTTCTCCAAAGAGGA.

### Conditioned Media

Wnt3a and control conditioned media were prepared from commercially available L cells stably transfected with a Wnt3a expression plasmid, or non-transfected control L cells (ATCC, USA), as previously described [8].

### Protein Isolation and Western Blot Analysis

Protein lysates from EBs were isolated in RIPA lysis buffer as previously described [8]. Protein lysates were separated on 8–10% SDS-PAGE gels as previously described (). Antibodies used included rabbit anti-pSmad1, rabbit anti-Smad1/5, rabbit anti-pSmad2, rabbit anti-Smad2/3, rabbit anti-pErk, rabbit anti-Erk, rabbit anti-p-p38, rabbit anti-p38, rabbit anti-Lrp6, rabbit anti-pLrp6 (all Cell Signaling Technology, USA), mouse anti-beta-catenin (Santa Cruz Biotechnology, USA), cardiac myosin heavy chain (Abcam, USA), anti-CS-E antibody GD3G7 [54], [55], [56], and mouse anti-alpha-tubulin (Santa Cruz Biotechnology, USA).

### TOPFLASH Reporter Assays

ES cells or EBs were transiently transfected with firefly TOPFLASH [49] and Renilla luciferase transfection control reporter constructs, using linear PEI (MW: 25,000; Polysciences, USA; PEI/DNA ratio of 8∶1) as a transfection reagent as previously described [8]. Three hours post-transfection the cells were stimulated with Wnt3a-CM or control L-CM for 24 hours. Dual luciferase assays were performed according to manufacturer’s instructions (Promega, USA; Biotium, USA).

### Statistical Analysis

Experiments were performed in triplicates; appropriate statistical analysis was conducted for comparisons among groups (Student’s t-test).

## Results

### CS Expression During ES Cell Differentiation and EB Culture

**Figure 1:**
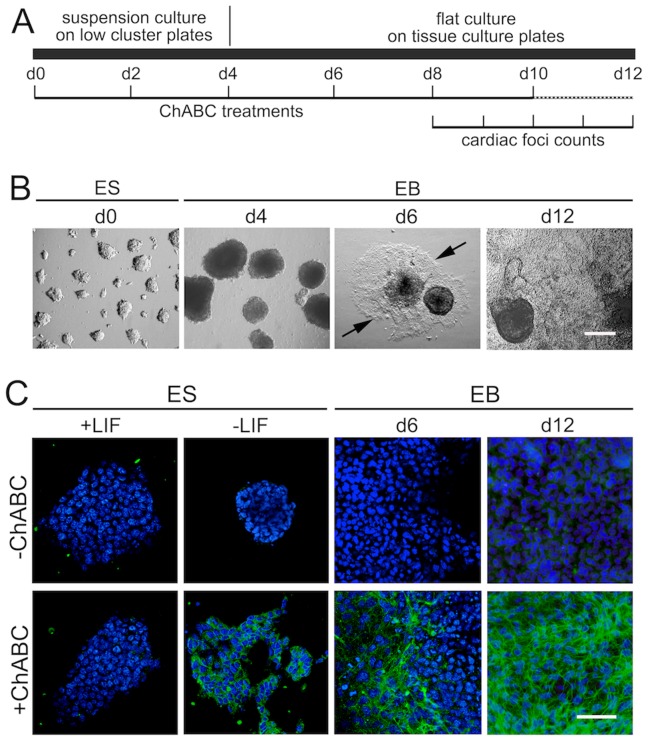
CS expression in differentiating ES cells and EB cultures. (A) Schematic of EB culture protocol. (B) Morphology of cultured cells: ES cells in the presence of LIF (d0); EBs in low cluster plates at day 4 (d4); EBs on tissue culture plates (d6; arrows point to forefront of cells migrating out of initial spheric EB), EBs at day 12 (d12). Scale bar = 200 micrometer. (C) Expression of CS in ES cells and EBs. No staining for CS stubs (green) was observed in the absence of ChABC treatment (top row). Bottom row: ChABC treatment of ES cells cultured in the presence of LIF also showed no CS expression. Differentiation of ES cells through withdrawal of LIF for 6 days led to CS expression. EBs at d6 of differentiation showed moderate CS expression, EBs at d12 of differentiation showed strong CS expression. Scale bar = 50 micrometer.

In order to investigate the roles of endogenous chondroitin sulfates in ES cell differentiation, we employed a previously established EB flat culture system [4]. R1 ES cells were cultured on low cluster plates for 4 days, and then transferred to tissue culture plates for another 8 days (Figure 1A). While undifferentiated ES cells grow in clumps with well-defined borders (d0; Figure 1B), withdrawal of LIF and differentiation in low cluster plates leads to large round unattached EB structures (d4; Figure 1B). Transfer to tissue culture plates leads to attachment and flattening of EB structures, with cells migrating out of the central stucture at d6 (Figure 1B), and EBs covering most or all of the well surface at d12 (Figure 1B). We then wanted to determine whether CS moieties are expressed in ES cells or EB cultures. For this, cultures were treated with the bacterial enzyme Chondroitinase ABC (ChABC) at 10 mU/ml, previously demonstrated to efficiently to eliminate chondroitin sulfate side chains of chondroitin sulfate proteoglycans (CSPGs) [48]. Subsequently, CS expression was visualized by immunofluorescence microscopy utilizing an antibody that recognizes the remaining CS stubs on CSPGs after ChABC digestion (Figure 1C), as described previously [12], [48]. As a control, cells were not treated with ChABC; indeed, no immunofluorescence signal was detected in ES cells cultured in the presence or absence of LIF, or in EBs at d6 or d12 of differentiation (Figure 1C). When treated with ChABC, undifferentiated ES (+LIF) cells showed no immunofluorescence signal, while differentiation by withdrawal of LIF for 6 days lead to expression of CS. Moreover, EB cultures at d6 and d12 showed increasing levels of CS (Figure 1C). These data suggested that CS side chains are not present at significant levels in undifferentiated ES cells, but become upregulated during differentiation.

### Elimination of CS in Differentiating ES Cells Affects Pluripotency Markers

**Figure 2:**
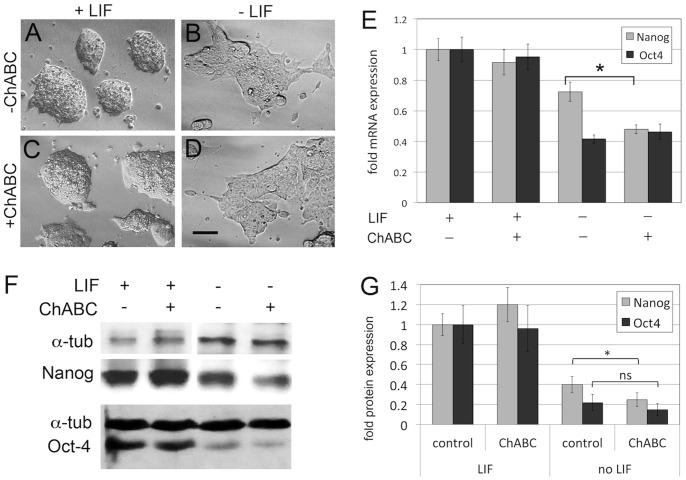
Enzymatic elimination of CS accelerates loss of *Nanog* expression in differentiating ES cells. (A–D) Morphology of ES cell cultures in the presence (A, C) or absence (B, D) of LIF, and in the presence (A,B) or absence (C,D) of ChABC. No major morphological changes were observed in ChABC-treated cultures. Scale bar = 20 micrometer. (E) Quantitation of expression of the pluripotency markers *Nanog* and *Oct4* by qPCR. In the presence of LIF, treatment of ES cells with ChABC did not alter expression of *Nanog* and *Oct4*. In the absence of LIF, expression of *Nanog* and *Oct4* was downregulated in differentiating ES cells, as expected. Treatment with ChABC lead to a further reduction in *Nanog* expression levels. (F) Western blot analysis of Nanog and Oct4 protein expression in the presence and absence of LIF and/or ChABC. Alpha-tubulin is shown as loading control. (G) Quantitation of three independent Western blots: ChABC did not affect protein levels in ES cell cultures in the presence of LIF. Withdrawal of LIF caused a reduction of Nanog and Oct4 protein levels, and concomitant treatment with ChABC caused an additional significant reduction in Nanog protein levels, while not significantly affecting Oct4 levels (*p<0.05).

We next analyzed the effect of ChABC treatment on ES cell morphology and expression of stem cell markers (Figure 2). While ES cells cultured in LIF formed round structures with even borders (Figure 2A), withdrawal of LIF for 6 days led to differentiation and altered morphology of ES cell cultures, with a more flattened appearance and uneven boundaries (Figure 2B), as previously described [4], [50]. Treatment with ChABC did not alter the overall appearance of ES cells either in the presence (Figure C) or absence (Figure 2D) of LIF. We then asked whether the expression of the known pluripotency markers *Nanog* and *Oct4* (also called *Pou5F1*) were affected by ChABC treatment (Figure 2E). Quantitative real time PCR (qPCR) demonstrated that in the presence of LIF, ES cells expressed high levels of *Nanog* and *Oct4*; these levels of expression were not altered by treatment with ChABC. This result is consistent with the lack of CS molecules in undifferentiated ES cells shown in Figure 1. Withdrawal of LIF lead to a reduction of both *Nanog* and *Oct4* expression (Figure 2E), as previously described. In this context, ChABC treatment did not affect Oct4 mRNA levels, but caused an additional significant reduction in *Nanog* mRNA levels (Figure 2E). We also used Western blot analysis (Figure 2F) to quantify Nanog and Oct4 protein levels (Figure 2G). Similar to our mRNA analysis, ChABC did not affect protein levels in ES cell cultures in the presence of LIF (Figure 2F,G). Withdrawal of LIF caused a reduction of Nanog and Oct4 protein levels, and concomitant treatment with ChABC caused an additional significant reduction in Nanog protein levels, while not significantly affecting Oct4 levels (Figure 2G). These results show that endogenous CS could affect the expression of some pluripotency markers in ES cells once differentiation has been initiated.

### CS Play a Role in Cardiac Differentiation of EB Cultures

**Figure 3:**
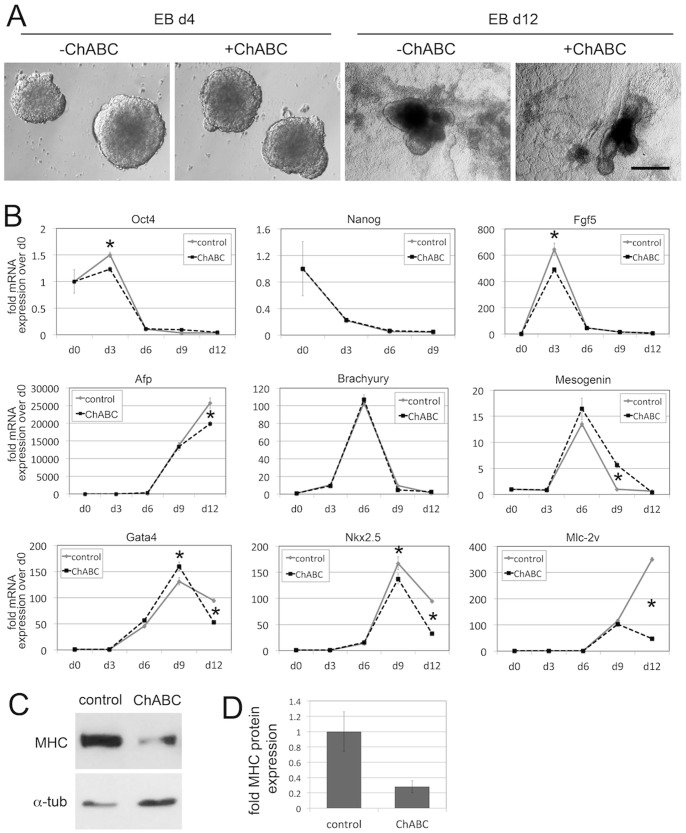
Lineage marker analysis in EB cultures reveals a role for endogenous CS in cardiac lineage development. (A) Morphology of EB cultures on d4 and d12 of differentiation, in the presence or absence of ChABC treatment. No morphological differences were observed with ChABC treatment. (B) Lineage marker analysis of EB cultures by qPCR. The pluripotency markers *Oct4* and *Nanog* severely decreased during differentiation, as expected. Treatment with ChABC did not affect the loss of *Oct4* and *Nanog*, apart from a temporary reduction of *Oct4* on d3. Expression of the early ectoderm marker *Fgf5* spiked on day 3 in control cultures, and then decreased drastically in later time points. Treatment with ChABC showed the same pattern, with somewhat reduced expression on day 3 when compared to controls. Expression of the endoderm marker *Afp* was initiated only after day 6, and rose steadily thereafter in control cultures. The same pattern was present with ChABC treatment; however, *Afp* expression was reduced by 20% on d12. Expression of the early and axial mesoderm marker *Brachyury* spiked on d6, and was indistinguishable between control and ChABC-treated cultures. *Mesogenin* as a marker of paraxial mesoderm also spikes at d6, but expression did not decline as fast as expression of *Brachyury*. Treatment with ChABC led to an increased expression of Mesogenin on d9, but was equivalent to controls on d12. Expression of the cardiac transcription factors *Gata4* and *Nkx2.5* started between d3 and d6 and peaked on d9, followed by a reduction on d12. Treatment with ChABC led to an approximately 50% reduction in *Gata4* and *Nkx2.5* expression on d12. Expression of structural cardiac marker *Mlc-2v* started only after d6, and increased dramatically by d12. EB cultures treated with ChABC displayed a severe reduction of *Mlc-2v* expression on d12 when compared to controls. (*p<0.05). (C,D) Western blot analysis of cardiac myosin heavy chain (MHC). MHC protein levels were significantly reduced by treatment of EBs with ChABC (p<0.05).

In the next set of experiments, we asked whether treatment with ChABC could identify a role for endogenous CS in the lineage-specific differentiation of EBs (Figure 3). For this, ES cells were differentiated in EB cultures, and treated bi-daily with ChABC, as indicated in Figure 1A. Treatment with ChABC efficiently digested CS chains in these cultures (Figure 1C). Elimination of CS did not affect the overall appearance of EB cultures on either day 4 or day 12 of differentiation (Figure 3A). We then prepared mRNA from EB cultures on days 0, 3, 6, 9 and 12 and analyzed expression of lineage markers by qPCR (Figure 3B). The pluripotency markers *Oct4* and *Nanog* severely decreased during differentiation, as expected. Treatment with ChABC did not affect the loss of *Oct4* and *Nanog* in this time course experiment, apart from a temporary reduction of *Oct4* on day 3. Expression of the early ectoderm marker *fibroblast growth factor-5* (*Fgf5*) spiked on day 3 in control cultures, and then decreased drastically in later time points. Treatment with ChABC showed the same pattern, with somewhat reduced expression on day 3 when compared to controls. Expression of the endoderm marker *alpha-fetoprotein* (*Afp*) was initiated only after day 6, and rose steadily thereafter in control cultures. The same pattern was present with ChABC treatment; however, *Afp* expression was reduced by 20% at day 12. Expression of the early and axial mesoderm marker *Brachyury* spiked on day 6, and was indistinguishable between control and ChABC-treated cultures. *Mesogenin* as a marker of paraxial mesoderm also spikes on day 6, but expression did not decline as fast as expression of *Brachyury*. Treatment with ChABC lead to an increased expression of Mesogenin on day 9, but was equivalent to controls on day 12. We next investigated markers of cardiac differentiation in our experimental setup. The transcription factors *Gata4* and *Nkx2.5* are early determinants of cardiac differentiation. Expression of these two factors started between days 3 and 6 and peaked on day 9, followed by a reduction on day 12 in control conditions. Interestingly, treatment with ChABC led to an approximately 50% reduction in *Gata4* and *Nkx2.5* expression on day 12 (Figure 3B). To corroborate these findings, we analyzed expression of *myosin light chain -2 ventricle* (*Mlc-2v*), a marker of differentiated cardiomyocytes. Expression started only after day 6, which is consistent with the onset of cardiomyocyte differentiation in these EB cultures, and increased dramatically on day 12 in control cultures. EB cultures treated with ChABC displayed a severe reduction of *Mlc-2v* expression on day 12 when compared to controls (Figure 3B). In order to independently verify this effect on cardiac lineage markers, we analyzed and quantified expression of cardiac myosin heavy chain protein (MHC) by Western blot analysis (Figure 3C,D). Indeed, MHC protein levels were significantly reduced by treatment of EBs with ChABC (Figure 3C,D). Together, these results showed that elimination of endogenous CS molecules affected the development of the cardiac lineage, while the development of other lineages appeared not significantly disturbed.

### Elimination of Endogenous CS Inhibits the Formation of Beating Cardiac Foci

**Figure 4:**
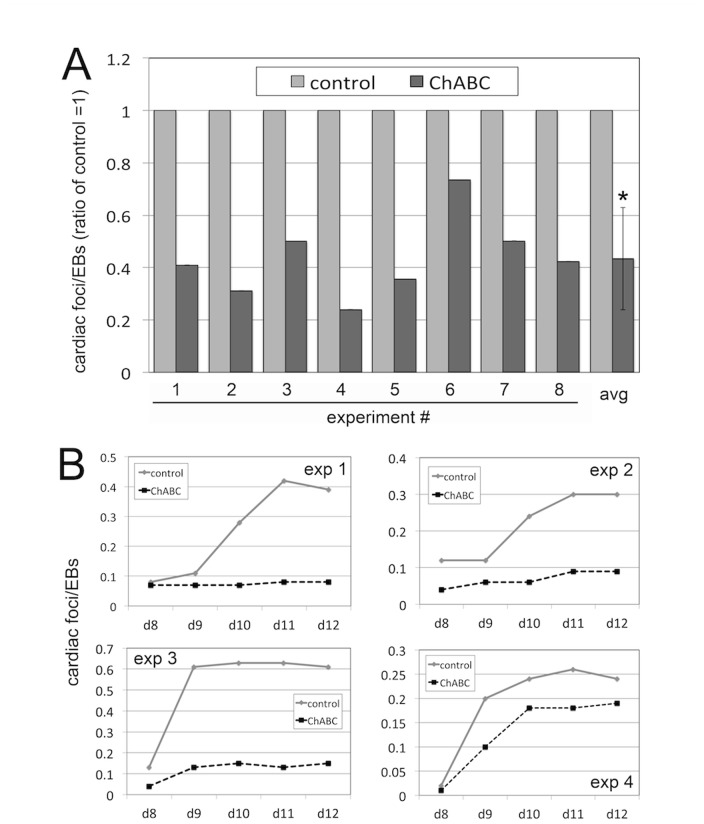
ChABC treatment reduces cardiac foci formation in EB cultures. (A) Ratios of beating cardiac foci corrected for total EBs plated in 8 independent experiments, and average ratio. Elimination of endogenous CS by ChABC led to a consistent, approximately 60% reduction in the number of beating cardiac foci. (*: p<0.05). (B) Time course of cardiac foci development in EB cultures. In 4 independent experiments, ChABC treatment lead to decreased numbers of cardiac foci even at d8 and d9, the earliest time points beating foci could be detected.

EB cultures have been shown to initiate and maintain the formation of functional cardiomyocytes into beating cardiac foci, which can be observed and quantified microscopically [51], [52]. Since ChABC treatment reduced expression of cardiomyocyte markers, we wanted to determine whether the development of functional beating cardiac foci was also affected. For this, we quantified the number of beating cardiac foci in relation to the number of EBs plated, in both control and EB cultures treated bi-daily with ChABC, in eight independent experiments (Figure 4A). Our data showed that bi-daily treatment with ChABC from day 0 to day 12 caused a consistent and significant reduction in the number of beating cardiac foci, with an average reduction of approximately 60% (Figure 4A). We then determined the temporal role of CS in cardiac foci formation in time course experiments (Figure 4B). In four independent experiments, we showed that the earliest time point beating foci were detectable was on day 8 of EB differentiation; in untreated cultures, the number of beating foci subsequently increased drastically, with a peak on day 11 to 12 (Figure 4B). Numbers of cardiac foci plateaued day 12 until day 15, and subsequently declined again (data not shown). Importantly, treatment with ChABC significantly decreased cardiac foci formation from the earliest time points on, when compared to control cultures (Figure 4B). Together with our data presented in Figure 3, these results indicated that the absence of endogenous CS interferes with the initial formation of beating cardiac foci through impaired cardiac differentiation.

### ChABC Treatment Activates the Wnt/Beta-catenin Pathway

**Figure 5:**
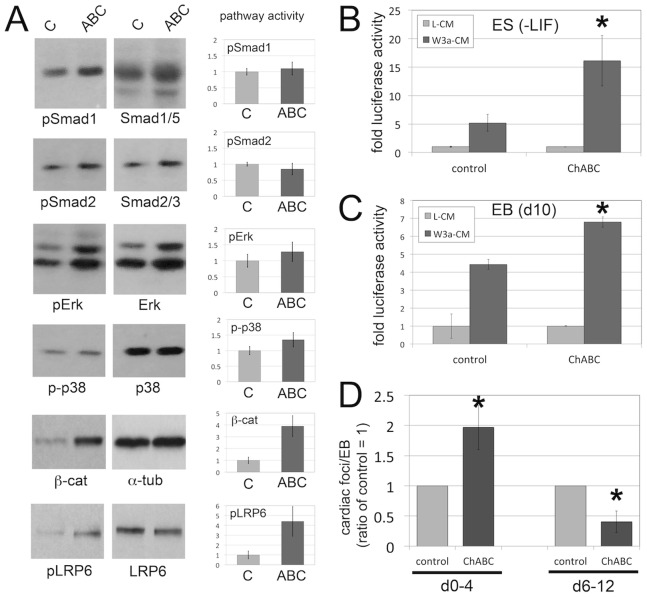
Elimination of CS leads to increased Wnt/beta-catenin signaling, and has a biphasic effect on cardiac foci formation. (A) Western blot analysis of TGFbeta, BMP, MAPK and Wnt/beta-catenin signaling pathways in control and ChABC-treated day 9 EB cultures (representative blots shown). No significant differences were detected in the levels of BMP signaling (levels of phosphorylated Smad1 (pSmad1) were corrected for total Smad1/5 levels), TGFbeta signaling (pSmad2 corrected for total Smad2 levels), Erk signaling (pErk corrected for total Erk levels), and p38 signaling (p-p38 corrected for total p38 levels). A significant induction of beta-catenin levels was observed with ChABC treatment (beta-catenin was corrected for total alpha-tubulin levels; p<0.05). Levels of pLRP6 were 5-fold increased in ChABC-treated EB cultures when compared to control cultures (p<0.05). Quantitation was performed on three independent experiments. (B,C) TOPFLASH luciferase reporter assays for Wnt/beta-catenin signaling activity. Transient transfection of the TOPFLASH reporter into ES cells cultured in the absence of LIF (B), or EB cultures (C), was followed by treatment with conditioned media containing Wnt3a ligand (W3a-CM), or control conditioned media (L-CM). Treatment with ChABC led to a significant increase in TOPFLASH reporter activity in both ES cells (B) and EBs (C). (D) Biphasic role of ChABC in cardiac foci formation. EB cultures were treated with ChABC either from d0 to d4, or from d6 to d12, followed by quantification of beating cardiac foci. Early treatment from d0 to d4 led to a significant increase in the numbers of cardiac foci, while late treatment (d6 to d12) caused a significant reduction in numbers of cardiac foci. (*: p<0.05).

In order to obtain a better mechanistic understanding of the observed roles of endogenous CS in cardiac differentiation, we analyzed the activities of several candidate signaling pathways known to affect cardiac development [53] (Figure 5). For this, we analyzed and quantified protein lysates of control and ChABC-treated EB cultures by western blot for activities of the TGFbeta, BMP, MAPK and Wnt/beta-catenin pathways (Figure 5A). No significant differences were detected in the levels of BMP signaling (levels of phosphorylated Smad1 (pSmad1) were corrected for total Smad1/5 levels), TGFbeta signaling (pSmad2 corrected for total Smad2/3 levels), Erk signaling (pErk corrected for total Erk levels), and p38 signaling (p-p38 corrected for total p38 levels). However, we observed a significant induction of beta-catenin levels with ChABC treatment (beta-catenin was corrected for total alpha-tubulin levels) (Figure 5A). This effect on Wnt/beta-catenin signaling was confirmed by western blot analysis of LRP6 receptor phosphorylation, a critical step in Wnt/beta-catenin pathway activation. Indeed, pLRP6 levels were 5-fold increased in ChABC-treated EB cultures when compared to control cultures (Figure 5A). These results indicated that endogenous CS play an important role in negatively controlling the Wnt/beta-catenin pathway at the receptor level, while not affecting TGFbeta, BMP, or MAPK pathways. We next wanted to corroborate this effect of ChABC treatment on Wnt/beta-catenin signaling by TOPFLASH luciferase reporter assays, a frequently used transcriptional readout for Wnt/beta-catenin signaling activity. Transient transfection of the TOPFLASH reporter into ES cells cultured in the absence of LIF, or EB cultures, was followed by treatment with conditioned media containing Wnt3a ligand (W3a-CM), or control conditioned media (L-CM). Treatment with ChABC led to a significant increase in TOPFLASH reporter activity in both ES cells (Figure 5B) and EBs (Figure 5C). Together, these results demonstrated that during EB differentiation, endogenous CS could negatively regulate Wnt/β-catenin signaling through impairment of Wnt receptor complex activation. Elimination of these endogenous CS leads to increased Wnt/beta-catenin signaling.

### Biphasic Role of Endogenous CS in Cardiac Differentiation

Wnt/beta-catenin signaling has previously been established to play a biphasic role in cardiac differentiation of EBs [44], [45], [46]. Specification of early cardiac progenitor cells is promoted, while later differentiation of progenitor cells into cardiomyocytes is repressed by Wnt/beta-catenin signaling [44], [45], [46]. Since we showed above that endogenous CS could negatively regulate the Wnt/beta-catenin pathway in EBs, we hypothesized that CS has a similar biphasic role in cardiac differentiation through its ability to regulate the Wnt/beta-catenin pathway. If this hypothesis is correct, we would expect that ChABC treatment only at early stages of EB differentiation might increase cardiac differentiation and cardiac foci formation, since it would activate a positive regulator of early cardiac progenitor development. Conversely, ChABC treatment only at later stages would be expected to decrease cardiac differentiation, since it would activate a now negative regulator of cardiomyocyte differentiation. For these experiments, EB cultures were treated with ChABC either from day 0 to day 4, or from day 6 to day 12, followed by quantification of cardiac foci formation (Figure 5D). Early treatment from day 0 to day 4 did indeed lead to a significant increase in cardiac foci formation, while late treatment from day 6 to day 12 caused a significant reduction in cardiac foci (Figure 5D). These results suggest that endogenous CS could mimic the known biphasic roles of Wnt/beta-catenin signaling during EB cardiac differentiation.

### Exogenous CS-E Can Negatively Regulate Wnt/Beta-catenin Signaling in EB Cultures

**Figure 6:**
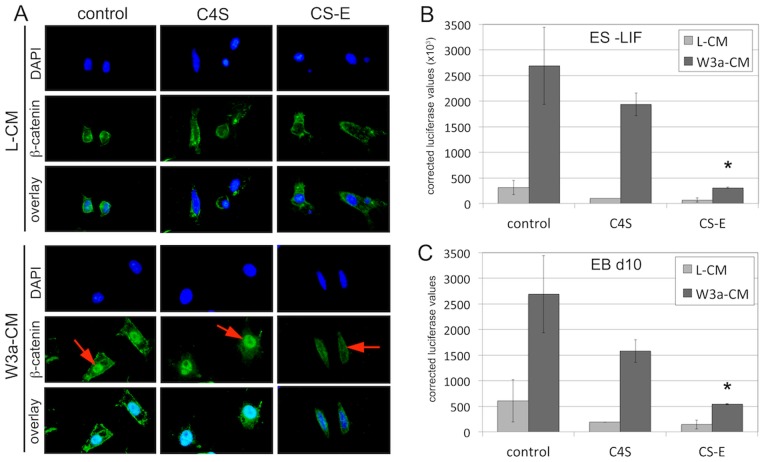
Treatment with exogenous CS-E represses Wnt/beta-catenin signaling in ES cells and EB cultures. (A) Immunofluorescence detection of beta-catenin (green) in ES cells in the absence of LIF (nuclei are stained blue with DAPI). Cells cultured in the absence of LIF and treated with the control L-CM did not show any nuclear beta-catenin, but low levels of membrane-bound beta-catenin; treatment with either C4S or CS-E did not alter levels or distribution of beta-catenin. When cultures were stimulated with W3a-CM, we observed a strong increase in nuclear accumulation of beta-catenin (red arrows). This nuclear accumulation was not altered by treatment with C4S, but was completely abolished by treatment with CS-E. Scale bar = 5 micrometer. (B,C) TOPFLASH reporter assays. In both ES cells cultured in the absence of LIF (B) and EBs on d10 of differentiation (C), treatment with exogenous CS-E could significantly inhibit TOPFLASH reporter activity induced by W3a-CM, while treatment with exogenous C4S had no significant effect. (*: p<0.05).

In this next set of experiments, we wanted to determine whether specific sulfation forms of chondroitin mediate the observed repressive effect on Wnt/beta-catenin signaling. We and others have recently shown that CS-E, but not other CS forms, can function as a potent inhibitor of the Wnt/beta-catenin pathway in fibroblasts [8], [47] and breast cancer cells (Willis and Klüppel, unpublished results). Thus, we initiated immunofluorescence studies to test whether treatment with exogenous CS-E at 100 microgramm/ml, previously shown to elicit a maximum inhibition of Wnt/beta-catenin signaling in NIH3T3 fibroblasts [8], could interfere with Wnt/beta-catenin nuclear accumulation in response to Wnt3a stimulation in differentiating ES cells (Figure 6A). As a control, ES cells were treated with exogenous C4S, a chondroitin sulfation form that has been shown not to affect Wnt/beta-catenin signaling [8], [47]. Control ES cells cultured in the absence of LIF and treated with the control L-CM did not show any nuclear beta-catenin, but low levels of membrane-bound beta-catenin; treatment with either C4S or CS-E did not alter the levels or distribution of beta-catenin. When cultures were stimulated with W3a-CM, we observed a strong increase in nuclear accumulation of beta-catenin. This nuclear accumulation was not altered by treatment with C4S, but was completely abolished by treatment with CS-E (Figure 6A). These results demonstrated that CS-E, but not C4S, could function as a potent inhibitor of beta-catenin activation in differentiating ES cells. We extended these findings by analyzing TOPFLASH reporter activities in either ES cells cultured in the absence of LIF (Figure 6B), or EBs at d10 of differentiation (Figure 6C). In both cases, treatment with exogenous CS-E could significantly inhibit TOPFLASH reporter activity induced by W3a-CM, while treatment with exogenous C4S had no significant effect (Figures 5B,C). Together, these results demonstrated that exogenous CS-E, but not C4S, could inhibit Wnt/beta-catenin signaling in differentiating ES cells and EB cultures.

### CS-E Expression in EB Cultures

**Figure 7:**
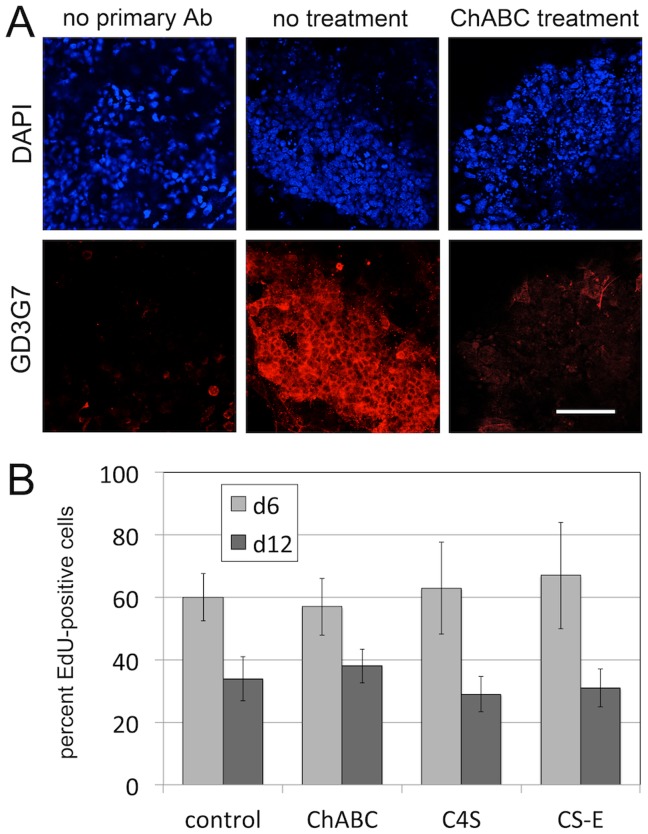
CS-E expression in EB cultures and proliferation. (A) The CS-E-specific antibody GD3G7 detected widespread expression of CS-E moieties (no treatment). In the absence of primary antibody (no primary Ab), or after treatment with ChABC (ChABC treatment), no specific signal was observed. (Scale bar = 100 micrometer). (B) Quantification of proliferation by EdU incorporation in EB cultures, in the presence and absence of treatments with ChABC, C4S and CS-E. At either d6 or d12 of EB cultures, elimination of endogenous CS by treatment with ChABC, or treatment with exogenous C4S or CS-E did not significantly alter the percentage of EdU-positive cells when compared to untreated controls.

Next, we wanted to determine whether endogenous CS-E is present in EB cultures. For this, cultures were fixed at d12 and subsequently incubated with the previously developed antibody GD3G7 specific for CS-E epitopes [54], [55], [56] (Figure 7A). GD3G7 detected widespread expression of CS-E moieties (Figure 7A, no treatment); in the absence of primary antibody (Figure 7A, no primary Ab), or after treatment with ChABC (Figure 7A, ChABC treatment), no specific signal was observed. These data demonstrated that CS-E-containing proteoglycans are expressed in our EB cultures.

### Chondroitin Sulfates do not Affect Proliferation of EB Cultures

We have shown above that chondroitin sulfates can control Wnt/beta-catenin signaling, a pathway that controls cell fate decisions as well as proliferative responses [35]. In order to determine whether our treatments affect proliferation of EBs, we analyzed and quantified incorporation of the nucleotide analog EdU in EB cultures, in the presence and absence of treatments with ChABC, C4S and CS-E. At either d6 or d12 of EB cultures, elimination of endogenous CS by treatment with ChABC, or treatment with exogenous C4S or CS-E did not significantly alter the percentage of EdU-positive cells when compared to untreated controls (Figure 7B). These results suggest that endogenous and exogenous chondroitin sulfates do not affect cell proliferation in our EB cultures.

### Biphasic Effect of Treatment with Exogenous CS-E on Cardiac Differentiation

**Figure 8:**
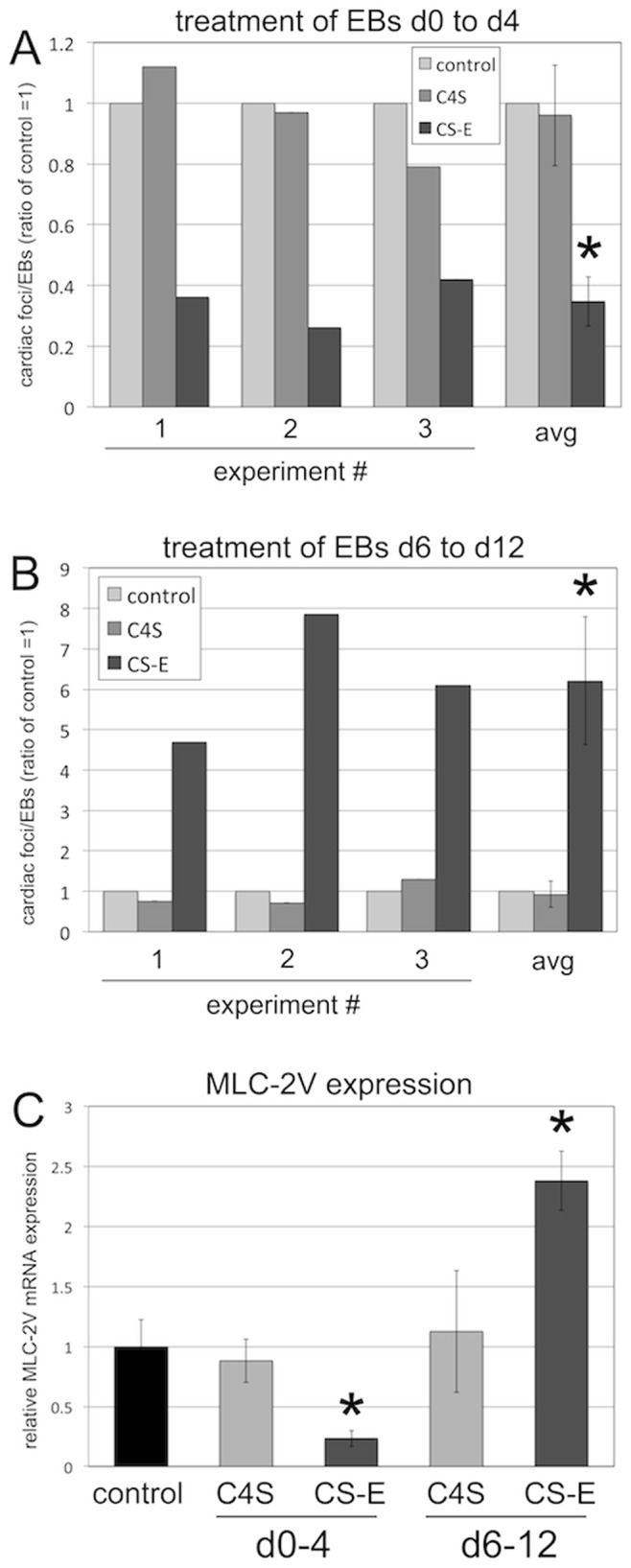
Treatment with exogenous CS-E has a biphasic role in cardiac foci development. (A) Treatment of EB cultures with exogenous CS-E, but not C4S, from d0 to d4 significantly decreased cardiac foci formation. Three independent experiments and average are shown. (* = p<0.05). (B) Treatment of EB cultures with exogenous CS-E, but not C4S, from d6 to d12 significantly increased cardiac foci formation. Three independent experiments and average are shown. (* = p<0.05). (C) Expression of *Mlc-2v* by qPCR. Treatment of EB cultures with exogenous CS-E, but not C4S, from d0 to d4 significantly decreased *Mlc-2v* expression. Treatment with exogenous CS-E, but not C4S, from d6 to d12 significantly increased *Mlc-2v* expression. (* = p<0.05).

We next set out to investigate whether treatment with exogenous CS-E could be used to drive cardiac differentiation of EB cultures, and whether CS-E treatment would display the same biphasic effects we observed with the elimination of endogenous CS. For this, EB cultures were treated with exogenous CS-E from either day 0 to day 4 (Figure 8A), or at later stages from day 6 to day 12 (Figure 8B). As a control, cultures were treated with C4S, which does not affect Wnt/beta-catenin signaling (Figure 6, and [8], [47]). We again hypothesized that treatment with CS-E at early stages of EB differentiation would decrease cardiac differentiation and cardiac foci formation, since CS-E could function as an inhibitor of Wnt/beta-catenin signaling. Conversely, CS-E treatment only at later stages would be expected to increase cardiac differentiation, since it would inhibit a pathway that now negatively regulates cardiomyocyte differentiation. Indeed, treatment of EB cultures with CS-E from day 0 to day 4 decreased cardiac foci formation (Figure 8A) and *Mlc-2v* expression (Figure 8C), while treatment with exogenous CS-E from day 6 to day 12 strongly increased cardiac foci formation (Figure 8B) and *Mlc-2v* expression (Figure 8C). In contrast, treatment with C4S did not affect cardiac foci formation by either early (Figure 8A) or late (Figure 8B) treatments, nor did C4S have any effect on *Mlc-2v* expression (Figure 8C). These results demonstrated that treatment with exogenous CS-E exhibited a biphasic role in cardiac differentiation, and that treatment at later stages of EB differentiation could be utilized to enhance cardiac differentiation.

## Discussion

**Figure 9:**
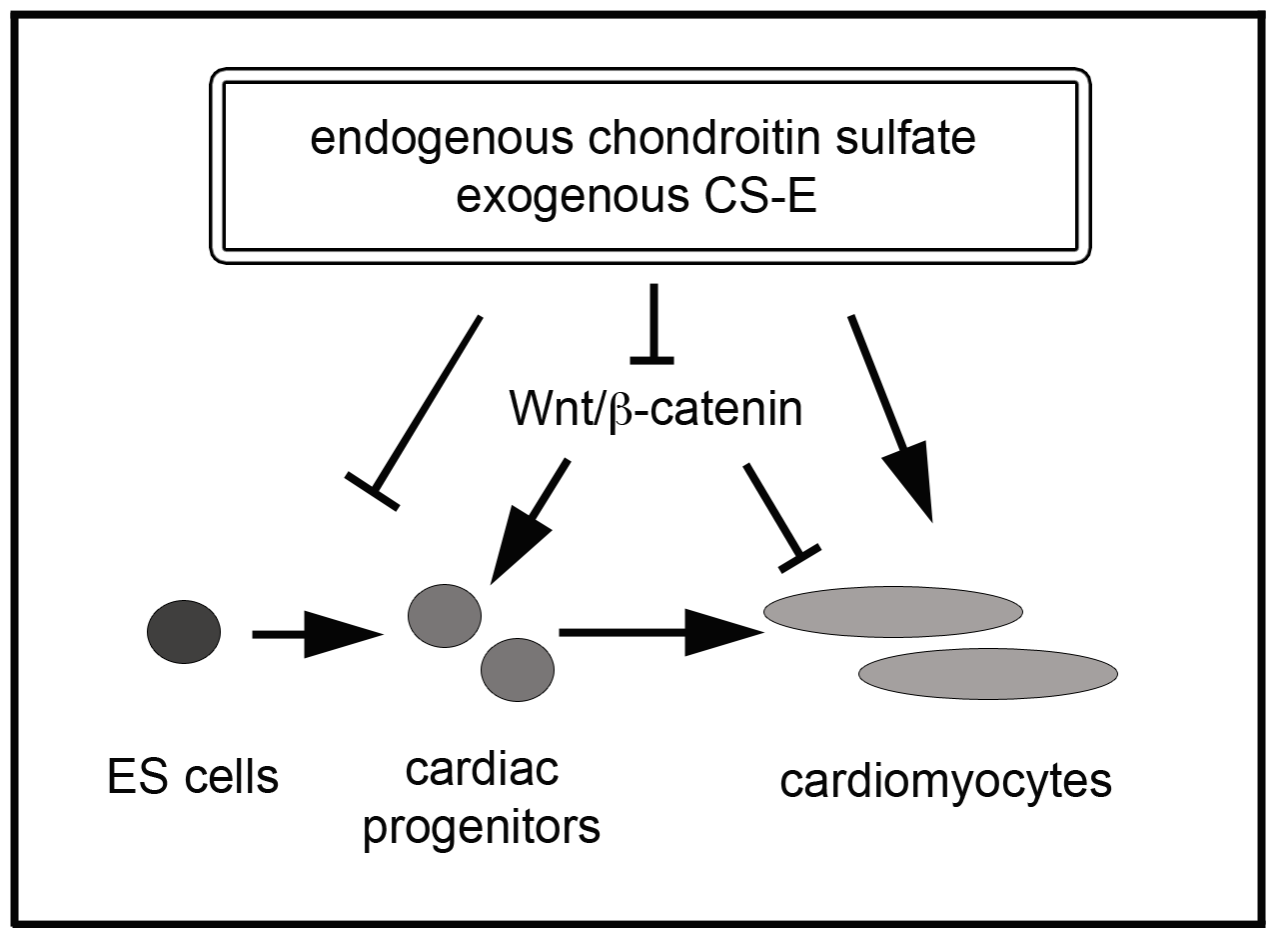
Model of the effects of CS on cardiac differentiation and Wnt/beta-catenin signaling. Endogenous CS and exogenous CS-E inhibit Wnt/beta-catenin signaling in EB cultures, leading to a reduction in early cardiac progenitors, and an enhancement of later differentiation steps into mature cardiomyocytes.

In this study, we describe a novel role of CS as biphasic regulators of cardiac differentiation of ES cells. Specifically, we show that elimination of endogenous CS at early stages promotes cardiac differentiation, whereas elimination of CS at later stages promotes cardiac differentiation. Treatment with exogenous CS-E could mimic these biphasic effects. Mechanistically, CS functioned through negative regulation of the Wnt/beta-catenin signaling cascade, in itself a known biphasic regulator of cardiac stem cell differentiation (Figure 9). This work represents the first study to shed light on the functional roles of CS in mammalian cardiac cell lineage determination. The phenotypic similarities between ChABC and CS-E treatments presented here suggest that CS-E might be the main endogenous CS in the control of cardiac differentiation through Wnt/beta-catenin signaling. This is supported by several lines of evidence: we show here that treatment with C4S did not show any of the effects we observed with CS-E treatments. Moreover, we demonstrate here and in previous work that CS-E, but not other forms of CS, could interfere with Wnt/beta-catenin signaling. However, we cannot exclude the possibility that chondroitin sulfate forms other than CS-E or C4S might contribute to cardiac differentiation by mechanisms independent from the control of Wnt/beta-catenin signaling. We also demonstrated that CS-E has a strong inhibitory effect on Wnt/beta-catenin signaling, a known regulator of cardiac differentiation. These results, however, do not exclude the possibility that CS-E also affects cardiac differentiation through mechanisms independent of its role as of Wnt/beta-catenin inhibitor. Future studies into the global roles of CS-E in cellular signaling events during cardiac differentiation will expand our knowledge in regards to the mechanistic actions of CS-E.

Several studies have highlighted cardiac expression domains of CS and enzymes involved in CS biosynthesis. For example, we showed previously that *C4st-1* is expressed in mouse embryonic heart from embryonic day 9 on, with initial expression in the atrioventricular canal, and strong expression in cardiac valves, the atrial myocardium and the outflow tract at later stages of embryogenesis [4]. A study in zebrafish has analyzed the roles of CS in heart development [57]. The authors showed that enzymatic and genetic ablation of CS in the developing heart caused defects in atrioventricular canal formation. Whether these results from zebrafish can be translated into the mammalian system is unknown. Interestingly, missense mutations in the human *Carbohydrate sulfotransferase 3* (*CHST3*) gene have been shown to be associated with Omani-type spondyloepiphyseal dysplasia. A subset of these patients exhibit mild cardiac abnormalities, including mitral, tricuspid and aortic regurgitations [26]. In contrast, *CHST3* knock-out mice and have neither skeletal nor cardiac defects [27]. Thus, the precise role of CHST3 in cardiac development and disease is not clear. A better mechanistic understanding of the roles of CS and its biosynthesis machinery in heart development is critical in order to better understand its role in cardiac disease and evaluate its potential as a pharmacological target.

While the roles of CS in mammalian heart development are not well understood, increasing evidence supports the notion that CS-containing proteoglycans (CSPGs) plays critical roles during cardiovascular injury. The CS side chains of the CSPG Biglycan can control elastin assembly in vascular walls [58], and targeted disruption of biglycan leads to abnormal collagen fibrils and aortic dissection and rupture [59]. Biglycan is also required for adaptive remodeling after myocardial infarction [60]. A recent study showed that CS molecules in myocardial scar tissue after ischemia-reperfusion-induced cardiac infarction could inhibit sympathetic nerve re-innervation into the injured area [61]. This was due to CS side chains on the CSPG protein tyrosine phosphatase sigma (PTPRS) in the injured region. This inhibitory role of CS on nerve regeneration appears similar to its roles in the glial scar in spinal cord injury. However, a role for CS in the regeneration of cardiomyocytes has not been established by this study. It will be interesting to determine whether the principle of a biphasic role of CS in cardiomyocyte differentiation identified here could be developed into a regenerative strategy in which a temporal modulation of CS levels and/or its sulfation balance, or treatment with exogenous CS-E, could promote stem cell differentiation into functional cardiomyocytes *in vitro* and/or *in vivo* after heart injury.

From a signaling point of view, this work has important pharmacological implications. Aberrantly activated Wnt/beta-catenin signaling has been associated in a number of human diseases and malignancies, including cancer, fibrosis, and cardiovascular disease [32], [33], [35], [36], [62], [63]. Thus, much effort has gone into the identification and characterization of Wnt pathway inhibitors, although translation of this research into clinical applications has not been successful thus far [64], [65], [66], [67]. Our previous results, together with the results presented here, suggest that the CS biosynthesis machinery could be an important pharmacological target to control aberrant Wnt/beta-catenin signaling in a number of biological and pathological systems, including cardiovascular disease.

## References

[1] Habuchi O (2000) Diversity and functions of glycosaminoglycan sulfotransferases. Biochim Biophys Acta 1474: 115–127. 10742590 10.1016/s0304-4165(00)00016-7

[2] Klüppel M (2010) The roles of chondroitin-4-sulfotransferase-1 in development and disease. Prog Mol Biol Transl Sci 93: 113–132. 20807643 10.1016/S1877-1173(10)93006-8

[3] Kusche-Gullberg M, Kjellen L (2003) Sulfotransferases in glycosaminoglycan biosynthesis. Curr Opin Struct Biol 13: 605–611. 14568616 10.1016/j.sbi.2003.08.002

[4] Klüppel M, Vallis KA, Wrana JL (2002) A high-throughput induction gene trap approach defines C4ST as a target of BMP signaling. Mech Dev 118: 77–89. 12351172 10.1016/s0925-4773(02)00198-3

[5] Willis CM, Wrana JL, Klüppel M (2009) Identification and characterization of TGFbeta-dependent and -independent cis-regulatory modules in the C4ST-1/CHST11 locus. Genet Mol Res 8: 1331–1343. 19937589 10.4238/vol8-4gmr673

[6] Klüppel M, Samavarchi-Tehrani P, Liu K, Wrana JL, Hinek A (2012) C4ST-1/CHST11-controlled chondroitin sulfation interferes with oncogenic HRAS signaling in Costello syndrome. European Journal of Human Genetics : EJHG 20: 870–877. 22317973 10.1038/ejhg.2012.12 PMC3400726

[7] Klüppel M, Wight TN, Chan C, Hinek A, Wrana JL (2005) Maintenance of chondroitin sulfation balance by chondroitin-4-sulfotransferase 1 is required for chondrocyte development and growth factor signaling during cartilage morphogenesis. Development 132: 3989–4003. 16079159 10.1242/dev.01948

[8] Willis CM, Klüppel M (2012) Inhibition by chondroitin sulfate E can specify functional Wnt/beta-catenin signaling thresholds in NIH3T3 fibroblasts. The Journal of Biological Chemistry 287: 37042–37056. 22915582 10.1074/jbc.M112.391490 PMC3481305

[9] Yu P, Pisitkun T, Wang G, Wang R, Katagiri Y, et al (2013) Global analysis of neuronal phosphoproteome regulation by chondroitin sulfate proteoglycans. PloS one 8: e59285. 23527152 10.1371/journal.pone.0059285 PMC3601063

[10] Mizumoto S, Fongmoon D, Sugahara K (2012) Interaction of chondroitin sulfate and dermatan sulfate from various biological sources with heparin-binding growth factors and cytokines. Glycoconjugate Journal. Aug 30(6): 619–32. 10.1007/s10719-012-9463-5 23275130

[11] Asimakopoulou AP, Theocharis AD, Tzanakakis GN, Karamanos NK (2008) The biological role of chondroitin sulfate in cancer and chondroitin-based anticancer agents. In Vivo 22: 385–389. 18610752

[12] Prinz RD, Willis CM, Viloria-Petit A, Klüppel M (2011) Elimination of breast tumor-associated chondroitin sulfate promotes metastasis. Genetics and molecular research : Genet Mol Res 10: 3901–3913. 10.4238/2011.December.8.9 22183949

[13] Theocharis AD, Theocharis DA, De Luca G, Hjerpe A, Karamanos NK (2002) Compositional and structural alterations of chondroitin and dermatan sulfates during the progression of atherosclerosis and aneurysmal dilatation of the human abdominal aorta. Biochimie 84: 667–674. 12453639 10.1016/s0300-9084(02)01428-1

[14] Theocharis AD, Tsara ME, Papageorgacopoulou N, Karavias DD, Theocharis DA (2000) Pancreatic carcinoma is characterized by elevated content of hyaluronan and chondroitin sulfate with altered disaccharide composition. Biochimica et Biophysica Acta 1502: 201–206. 11040445 10.1016/s0925-4439(00)00051-x

[15] Theocharis AD, Tsolakis I, Tzanakakis GN, Karamanos NK (2006) Chondroitin sulfate as a key molecule in the development of atherosclerosis and cancer progression. Advances in pharmacology 53: 281–295. 17239771 10.1016/S1054-3589(05)53013-8

[16] Uebelhart D (2008) Clinical review of chondroitin sulfate in osteoarthritis. Osteoarthritis Cartilage 16 Suppl 3S19–21. 18674931 10.1016/j.joca.2008.06.006

[17] Carulli D, Laabs T, Geller HM, Fawcett JW (2005) Chondroitin sulfate proteoglycans in neural development and regeneration. Current Opinion in Neurobiology 15: 116–120. 15721753 10.1016/j.conb.2005.01.014

[18] Bartus K, James ND, Bosch KD, Bradbury EJ (2012) Chondroitin sulphate proteoglycans: key modulators of spinal cord and brain plasticity. Experimental Neurology 235: 5–17. 21871887 10.1016/j.expneurol.2011.08.008

[19] Bradbury EJ, Carter LM (2011) Manipulating the glial scar: chondroitinase ABC as a therapy for spinal cord injury. Brain Research Bulletin 84: 306–316. 20620201 10.1016/j.brainresbull.2010.06.015

[20] Wang H, Katagiri Y, McCann TE, Unsworth E, Goldsmith P, et al (2008) Chondroitin-4-sulfation negatively regulates axonal guidance and growth. Journal of Cell Science 121: 3083–3091. 18768934 10.1242/jcs.032649 PMC2562295

[21] Purushothaman A, Sugahara K, Faissner A (2012) Chondroitin sulfate “wobble motifs” modulate maintenance and differentiation of neural stem cells and their progeny. The Journal of Biological Chemistry 287: 2935–2942. 22094467 10.1074/jbc.R111.298430 PMC3270950

[22] Wilson DG, Phamluong K, Lin WY, Barck K, Carano RA, et al (2012) Chondroitin sulfate synthase 1 (Chsy1) is required for bone development and digit patterning. Developmental Biology 363: 413–425. 22280990 10.1016/j.ydbio.2012.01.005

[23] Sato T, Kudo T, Ikehara Y, Ogawa H, Hirano T, et al (2011) Chondroitin sulfate N-acetylgalactosaminyltransferase 1 is necessary for normal endochondral ossification and aggrecan metabolism. The Journal of Biological Chemistry 286: 5803–5812. 21148564 10.1074/jbc.M110.159244 PMC3037693

[24] Hermanns P, Unger S, Rossi A, Perez-Aytes A, Cortina H, et al (2008) Congenital joint dislocations caused by carbohydrate sulfotransferase 3 deficiency in recessive Larsen syndrome and humero-spinal dysostosis. American Journal of Human Genetics 82: 1368–1374. 18513679 10.1016/j.ajhg.2008.05.006 PMC2427316

[25] Thiele H, Sakano M, Kitagawa H, Sugahara K, Rajab A, et al (2004) Loss of chondroitin 6-O-sulfotransferase-1 function results in severe human chondrodysplasia with progressive spinal involvement. Proceedings of the National Academy of Sciences of the United States of America 101: 10155–10160. 15215498 10.1073/pnas.0400334101 PMC454181

[26] Tuysuz B, Mizumoto S, Sugahara K, Celebi A, Mundlos S, et al (2009) Omani-type spondyloepiphyseal dysplasia with cardiac involvement caused by a missense mutation in CHST3. Clinical Genetics 75: 375–383. 19320654 10.1111/j.1399-0004.2009.01167.x

[27] Uchimura K, Kadomatsu K, Nishimura H, Muramatsu H, Nakamura E, et al (2002) Functional analysis of the chondroitin 6-sulfotransferase gene in relation to lymphocyte subpopulations, brain development, and oversulfated chondroitin sulfates. The Journal of Biological Chemistry 277: 1443–1450. 11696535 10.1074/jbc.M104719200

[28] Bashey RI, Sampson PM, Jimenez SA, Heimer R (1993) Glycosaminoglycans and chondroitin/dermatan sulfate proteoglycans in the myocardium of a non-human primate. Matrix 13: 363–371. 8246832 10.1016/s0934-8832(11)80041-7

[29] Hinek A, Teitell MA, Schoyer L, Allen W, Gripp KW, et al (2005) Myocardial storage of chondroitin sulfate-containing moieties in Costello syndrome patients with severe hypertrophic cardiomyopathy. American Journal of Medical Genetics Part A 133A: 1–12. 15637729 10.1002/ajmg.a.30495

[30] Behrens J (2005) The role of the Wnt signalling pathway in colorectal tumorigenesis. Biochem Soc Trans 33: 672–675. 16042571 10.1042/BST0330672

[31] Cadigan KM, Peifer M (2009) Wnt signaling from development to disease: insights from model systems. Cold Spring Harb Perspect Biol 1: a002881. 20066091 10.1101/cshperspect.a002881 PMC2742092

[32] Clevers H (2006) Wnt/beta-catenin signaling in development and disease. Cell 127: 469–480. 17081971 10.1016/j.cell.2006.10.018

[33] Fodde R, Brabletz T (2007) Wnt/beta-catenin signaling in cancer stemness and malignant behavior. Curr Opin Cell Biol 19: 150–158. 17306971 10.1016/j.ceb.2007.02.007

[34] Moon RT (2005) Wnt/beta-catenin pathway. Sci STKE 2005: cm1. 15713948 10.1126/stke.2712005cm1

[35] Nusse R (2005) Wnt signaling in disease and in development. Cell Res 15: 28–32. 15686623 10.1038/sj.cr.7290260

[36] Polakis P (2007) The many ways of Wnt in cancer. Curr Opin Genet Dev 17: 45–51. 17208432 10.1016/j.gde.2006.12.007

[37] Reya T, Clevers H (2005) Wnt signalling in stem cells and cancer. Nature 434: 843–850. 15829953 10.1038/nature03319

[38] Fevr T, Robine S, Louvard D, Huelsken J (2007) Wnt/beta-catenin is essential for intestinal homeostasis and maintenance of intestinal stem cells. Mol Cell Biol 27: 7551–7559. 17785439 10.1128/MCB.01034-07 PMC2169070

[39] Gaspar C, Fodde R (2004) APC dosage effects in tumorigenesis and stem cell differentiation. Int J Dev Biol 48: 377–386. 15349813 10.1387/ijdb.041807cg

[40] Huelsken J, Held W (2009) Canonical Wnt signaling plays essential roles. Eur J Immunol 39: 3582–3583; author reply 3583–3584. 10.1002/eji.200838982 19941311

[41] Nusse R, Fuerer C, Ching W, Harnish K, Logan C, et al (2008) Wnt signaling and stem cell control. Cold Spring Harb Symp Quant Biol 73: 59–66. 19028988 10.1101/sqb.2008.73.035

[42] Hirata A, Utikal J, Yamashita S, Aoki H, Watanabe A, et al (2013) Dose-dependent roles for canonical Wnt signalling in de novo crypt formation and cell cycle properties of the colonic epithelium. Development 140: 66–75. 23222438 10.1242/dev.084103 PMC3513993

[43] Kühl SJ, Kühl M (2013) On the role of Wnt/beta-catenin signaling in stem cells. Biochimica et biophysica acta 1830: 2297–2306. 22986148 10.1016/j.bbagen.2012.08.010

[44] Ueno S, Weidinger G, Osugi T, Kohn AD, Golob JL, et al (2007) Biphasic role for Wnt/beta-catenin signaling in cardiac specification in zebrafish and embryonic stem cells. Proceedings of the National Academy of Sciences of the United States of America 104: 9685–9690. 17522258 10.1073/pnas.0702859104 PMC1876428

[45] Gessert S, Kühl M (2010) The multiple phases and faces of wnt signaling during cardiac differentiation and development. Circulation Research 107: 186–199. 20651295 10.1161/CIRCRESAHA.110.221531

[46] Kwon C, Cordes KR, Srivastava D (2008) Wnt/beta-catenin signaling acts at multiple developmental stages to promote mammalian cardiogenesis. Cell cycle 7: 3815–3818. 19066459 10.4161/cc.7.24.7189 PMC2727475

[47] Nadanaka S, Ishida M, Ikegami M, Kitagawa H (2008) Chondroitin 4-O-sulfotransferase-1 modulates Wnt-3a signaling through control of E disaccharide expression of chondroitin sulfate. J Biol Chem 283: 27333–27343. 18667431 10.1074/jbc.M802997200

[48] Klüppel M (2011) Efficient secretion of biologically active Chondroitinase ABC from mammalian cells in the absence of an N-terminal signal peptide. Mol Cell Biochem 351: 1–11. 21213020 10.1007/s11010-010-0705-1

[49] Molenaar M, van de Wetering M, Oosterwegel M, Peterson-Maduro J, Godsave S, et al (1996) XTcf-3 transcription factor mediates beta-catenin-induced axis formation in Xenopus embryos. Cell 86: 391–399. 8756721 10.1016/s0092-8674(00)80112-9

[50] Nagy A, Gertsenstein M, Vintersten K, Behringer R (2006) Differentiating Embryonic Stem (ES) Cells into Embryoid Bodies. CSH protocols 2006. 10.1101/pdb.prot4405 22485815

[51] Dell’Era P, Ronca R, Coco L, Nicoli S, Metra M, et al (2003) Fibroblast growth factor receptor-1 is essential for in vitro cardiomyocyte development. Circulation research 93: 414–420. 12893744 10.1161/01.RES.0000089460.12061.E1

[52] Ronca R, Gualandi L, Crescini E, Calza S, Presta M, et al (2009) Fibroblast growth factor receptor-1 phosphorylation requirement for cardiomyocyte differentiation in murine embryonic stem cells. Journal of Cellular and Molecular Medicine 13: 1489–1498. 19549074 10.1111/j.1582-4934.2009.00805.x PMC3828861

[53] Chen K, Wu L, Wang ZZ (2008) Extrinsic regulation of cardiomyocyte differentiation of embryonic stem cells. Journal of Cellular Biochemistry 104: 119–128. 17979183 10.1002/jcb.21604

[54] ten Dam GB, van de Westerlo EM, Purushothaman A, Stan RV, Bulten J, et al (2007) Antibody GD3G7 selected against embryonic glycosaminoglycans defines chondroitin sulfate-E domains highly up-regulated in ovarian cancer and involved in vascular endothelial growth factor binding. The American Journal of Pathology 171: 1324–1333. 17717144 10.2353/ajpath.2007.070111 PMC1988881

[55] Purushothaman A, Fukuda J, Mizumoto S, ten Dam GB, van Kuppevelt TH, et al (2007) Functions of chondroitin sulfate/dermatan sulfate chains in brain development. Critical roles of E and iE disaccharide units recognized by a single chain antibody GD3G7. The Journal of Biological Chemistry 282: 19442–19452. 17500059 10.1074/jbc.M700630200

[56] Smetsers TF, van de Westerlo EM, ten Dam GB, Overes IM, Schalkwijk J, et al (2004) Human single-chain antibodies reactive with native chondroitin sulfate detect chondroitin sulfate alterations in melanoma and psoriasis. The Journal of Investigative Dermatology 122: 707–716. 15086557 10.1111/j.0022-202X.2004.22316.x

[57] Peal DS, Burns CG, Macrae CA, Milan D (2009) Chondroitin sulfate expression is required for cardiac atrioventricular canal formation. Developmental dynamics : an official publication of the American Association of Anatomists 238: 3103–3110. 19890913 10.1002/dvdy.22154 PMC2852642

[58] Hwang JY, Johnson PY, Braun KR, Hinek A, Fischer JW, et al (2008) Retrovirally mediated overexpression of glycosaminoglycan-deficient biglycan in arterial smooth muscle cells induces tropoelastin synthesis and elastic fiber formation in vitro and in neointimae after vascular injury. The American Journal of Pathology 173: 1919–1928. 18988796 10.2353/ajpath.2008.070875 PMC2626402

[59] Heegaard AM, Corsi A, Danielsen CC, Nielsen KL, Jorgensen HL, et al (2007) Biglycan deficiency causes spontaneous aortic dissection and rupture in mice. Circulation 115: 2731–2738. 17502576 10.1161/CIRCULATIONAHA.106.653980

[60] Westermann D, Mersmann J, Melchior A, Freudenberger T, Petrik C, et al (2008) Biglycan is required for adaptive remodeling after myocardial infarction. Circulation 117: 1269–1276. 18299507 10.1161/CIRCULATIONAHA.107.714147

[61] Gardner RT, Habecker BA (2013) Infarct-derived chondroitin sulfate proteoglycans prevent sympathetic reinnervation after cardiac ischemia-reperfusion injury. The Journal of neuroscience : the official journal of the Society for Neuroscience 33: 7175–7183. 23616527 10.1523/JNEUROSCI.5866-12.2013 PMC3671889

[62] Lam AP, Gottardi CJ (2011) beta-catenin signaling: a novel mediator of fibrosis and potential therapeutic target. Current opinion in rheumatology 23: 562–567. 21885974 10.1097/BOR.0b013e32834b3309 PMC3280691

[63] Oerlemans MI, Goumans MJ, van Middelaar B, Clevers H, Doevendans PA, et al (2010) Active Wnt signaling in response to cardiac injury. Basic Res Cardiol 105: 631–641. 20373104 10.1007/s00395-010-0100-9 PMC2916122

[64] Barker N, Clevers H (2006) Mining the Wnt pathway for cancer therapeutics. Nat Rev Drug Discov 5: 997–1014. 17139285 10.1038/nrd2154

[65] Prosperi JR, Goss KH (2010) A Wnt-ow of opportunity: targeting the Wnt/beta-catenin pathway in breast cancer. Curr Drug Targets 11: 1074–1088. 20545611 10.2174/138945010792006780

[66] Takahashi-Yanaga F, Kahn M (2010) Targeting Wnt signaling: can we safely eradicate cancer stem cells? Clin Cancer Res 16: 3153–3162. 20530697 10.1158/1078-0432.CCR-09-2943

[67] van Es JH, Clevers H (2005) Notch and Wnt inhibitors as potential new drugs for intestinal neoplastic disease. Trends Mol Med 11: 496–502. 16214417 10.1016/j.molmed.2005.09.008

